# Supplementary material for: Identification of risk factors for mortality associated with COVID-19
Source: PeerJ. 2020 Sep 1;8:e9885. doi: 10.7717/peerj.9885 (PMC7473053; doi:10.7717/peerj.9885)
Supplement: Supplemental Information 1 [file peerj-08-9885-s001.docx]

Supplemental digital content for early prediction of mortality in COVID-19 patients: development of neural network models by genetic algorithms

**Table of Contents**

[***Supplemental Method 2***](#_Toc34989766)

[**Genetic algorithm 2**](#_Toc34989767)

[**Neural network training 3**](#_Toc34989768)

[**Model interpretation with LIME 3**](#_Toc34989769)

[***eTable 1 Baseline characteristics between survivors and non-survivors on hospital discharge 5***](#_Toc34989770)

[***eFigure 1. Heatmap showing the difference between survival and non-survival groups. 10***](#_Toc34989771)

[***eFigure 2. Forward selection using the most frequent genes (variables). 11***](#_Toc34989772)

[***eFigure 3. Heatmap for NNet1 comprising 9 variables. 12***](#_Toc34989773)

[***eFigure 4. Heatmap for NNet1 comprising 32 variables. 13***](#_Toc34989774)

[***eFigure 5. Depiction of NNet1 in PCA space. 14***](#_Toc34989775)

[***eFigure 6. Depiction of NNet2 in PCA space. 15***](#_Toc34989776)

[***eFigure 7. Grid search hyperparameter tuning for NNet1 (A) and NNet2 (B). 16***](#_Toc34989777)

[***eFigure 8. Facetted heatmap-style visualization of all case-feature combinations for four selected patients. 17***](#_Toc34989778)

Supplemental Method

Genetic algorithm

The genetic algorithm is a type of evolutionary computer algorithm in which symbols (often called “genes” or “chromosomes”) representing possible solutions are “bred.” This “breeding” of symbols typically includes the use of a mechanism analogous to the crossing-over process in genetic recombination and an adjustable mutation rate. A fitness function is used on each generation of algorithms to gradually improve the solutions in analogy to the process of natural selection {Tolvi:2004cq, Trevino:2006if}. The process of evolving the GA and automating the selection is known as genetic programming. In the present study, each clinical variable was a gene; they formed a chromosome that is a combination of variables. The fitness function is the accuracy: the correctly predicted samples divided by the total samples. The GA was carried out with the following stages:

Stage 1: The procedure initially creates a number of random variable sets (5 variables/genes). These variable sets form a population of chromosomes (niche, a combination of variables).

Stage 2: Each chromosome in the population is evaluated for its ability to

predict the group membership of each sample in the dataset. The fitness function is the neural network model in our study. Specifically, a neural network model was trained by using 5 randomly selected variables, and the response variable was the in-hospital mortality. The GA tests the accuracy of the prediction and assigns a score to each chromosome that is proportional to the accuracy resulting in the fitness function.

Stage 3: When a chromosome has a score higher than 0.90, this chromosome is selected, and the procedure stops; otherwise, the procedure continues to stage 4.

Stage 4: The population of chromosomes is replicated. Chromosomes with a higher fitness score will generate more offspring.

Stage 5: The genetic information contained in the replicated parent chromosomes is combined through genetic crossover. Two randomly selected parent chromosomes are used to create two new chromosomes. This crossover mechanism allows a better exploration of possible solutions recombining good chromosomes.

Stage 6: Mutations are then introduced in the chromosome randomly. These mutations produce new genes that are used in chromosomes.

Stage 7: The process is repeated from stage 2 until an accurate chromosome is

obtained. The cycle of replication (stage 4), genetic cross‐over (stage 5) and mutation (stage 6) is called generation.

Neural network training

The hyperparameters of the neural network model included bagging, weight decay and the number of hidden units. The hyperparameter was chosen by the grid search method. The number of hidden units was 2, 4, 6, 8 and 10, the weight decay was 0.001, 0.01, 0.02 and 0.03, and the bagging was 100, 200 and 500. Two neural networks named NNet1 and NNet2 were trained in the study. NNet1 included 9 predictors whose predictive accuracy was higher than 99% of the maximum. NNet2 included 32 predictors whose predictive accuracy was the maximum. Five-fold cross-validation was employed to train the model. Cross-validation is a resampling procedure used to evaluate machine learning models on a limited data sample. The procedure has a single parameter called (i.e., k = 5 in our example) that refers to the number of groups that a given data sample should be split into. Cross-validation is commonly used in applied machine learning to estimate the skill of a machine learning model on unseen data. That is, a limited sample is used to estimate how the model is expected to perform in general when making predictions on data not used during model training {Stefaniak:2005wa, Wu:2016ff}.

The training process was performed in the following steps:

1. Shuffle the dataset randomly.
2. Split the dataset into k = 5 groups.
3. For each unique group (loop through 5 groups):
   1. Take the group as a hold out or test data set
   2. Take the remaining groups as a training data set
   3. Fit a neural network model on the training set and evaluate it on the test set
   4. Retain the evaluation score and discard the model
4. Summarize the prediction accuracy of the model using the sample of model evaluation scores

Model interpretation with LIME

Although neural network models can significantly improve prediction accuracy, their interpretation is an important shortcoming. This limitation is well known as an obstacle prohibiting the application of machine learning models to real clinical practice {Chen:2017ce}. Local interpretable model-agnostic explanations (LIME) has been widely used for the interpretation of neural networks and other black-box models.

The intuition behind LIME is that the behaviour of a complex model can be learned by perturbing the input and determining how the predictions change. This turns out to be a benefit in terms of interpretability because we can perturb the input by changing components that make sense to humans (e.g., a small change in LDH or CRP), even if the model is using much more complicated components as features (e.g., high-order interaction terms). We generated an explanation by approximating the neural network model with an interpretable one (such as a linear model with only a few non-zero coefficients) learned on perturbations of the original instance (e.g., change in LDH). The key intuition behind LIME is that it is much easier to approximate a black-box model by a simple model locally (in the neighbourhood of the prediction we want to explain) than to try to approximate a model globally. The R package lime was used in our study {Pedersen:2018wb, Zhang:2018cu}.

# eTable 1 Baseline characteristics between survivors and non-survivors on hospital discharge

| Variables | Total (n = 246) | survival  (n = 204) | Non-survival (n = 42) | p |
| --- | --- | --- | --- | --- |
| Age, Median (IQR) | 56.5 (43.25, 65) | 55 (41, 63) | 68.5 (56.75, 76.75) | < 0.001 |
| Sex, n (%) |  |  |  | 0.284 |
| Female | 121 (49) | 104 (51) | 17 (40) |  |
| Male | 125 (51) | 100 (49) | 25 (60) |  |
| LDL, Mean ± SD | 2.26 ± 0.64 | 2.28 ± 0.61 | 2.19 ± 0.81 | 0.547 |
| TC, Mean ± SD | 3.71 ± 0.81 | 3.73 ± 0.74 | 3.62 ± 1.08 | 0.52 |
| TG, Median (IQR) | 1.15 (0.94, 1.61) | 1.11 (0.93, 1.61) | 1.29 (0.99, 1.7) | 0.118 |
| High-sensitive cardiac troponin I, Median (IQR) | 0 (0, 0.01) | 0 (0, 0.01) | 0.03 (0, 0.06) | < 0.001 |
| HDL, Median (IQR) | 0.89 (0.73, 1.03) | 0.89 (0.75, 1.03) | 0.83 (0.71, 0.98) | 0.083 |
| nucleotidase, Median (IQR) | 1.96 (1.44, 3.35) | 1.96 (1.45, 3.12) | 2.13 (1.24, 3.45) | 0.756 |
| C-reactive protein, Median (IQR) | 34.77 (11.32, 74.89) | 23.49 (10.1, 53.59) | 85.75 (57.39, 164.65) | < 0.001 |
| D-dimer, Median (IQR) | 0.55 (0.31, 1.06) | 0.52 (0.26, 0.96) | 0.99 (0.44, 2.96) | < 0.001 |
| alpha-hydroxybutyrate dehydrogenase, Median (IQR) | 211.5 (164, 277.5) | 194.5 (160.75, 247.5) | 306.5 (268.75, 377.25) | < 0.001 |
| γ-glutamyltranspeptidase, Median (IQR) | 25.5 (16, 44.75) | 24 (15, 43.25) | 27 (19.25, 70.25) | 0.145 |
| Neutrophil percentage, Median (IQR) | 68.75 (60.6, 77.77) | 67.25 (59.5, 75.23) | 80.85 (69.98, 90.1) | < 0.001 |
| Neutrophil count, Median (IQR) | 3.26 (2.09, 4.67) | 3.08 (2.07, 4.04) | 5.02 (3.02, 7.95) | < 0.001 |
| lactate, Mean ± SD | 2.9 ± 0.97 | 2.85 ± 0.92 | 3.11 ± 1.16 | 0.183 |
| Lactate dehydrogenase, Median (IQR) | 266.5 (208.5, 349.75) | 241 (198.5, 317.25) | 381.5 (336.5, 464.25) | < 0.001 |
| Prothrombin time, Median (IQR) | 11.9 (11.4, 12.4) | 11.8 (11.3, 12.33) | 12.15 (11.5, 12.6) | 0.022 |
| Thrombin time, Median (IQR) | 20.2 (18.52, 22.55) | 20.5 (18.67, 22.8) | 19.2 (18.35, 21.2) | 0.017 |
| prealbumin, Median (IQR) | 135.95 (104.48, 181.75) | 141.1 (107.7, 186.9) | 106.2 (78.6, 147.17) | < 0.001 |
| Percentage of monocytes, Mean ± SD | 7.43 ± 3.21 | 7.78 ± 3.06 | 5.75 ± 3.43 | < 0.001 |
| Monocytes count, Median (IQR) | 0.35 (0.24, 0.47) | 0.36 (0.25, 0.47) | 0.31 (0.23, 0.41) | 0.221 |
| Percentage of basophils, Median (IQR) | 0.1 (0, 0.2) | 0.1 (0, 0.23) | 0 (0, 0.1) | < 0.001 |
| Basophils count, n (%) |  |  |  | 0.102 |
| 0 | 114 (46) | 86 (42) | 28 (67) |  |
| 0.01 | 93 (38) | 83 (41) | 10 (24) |  |
| 0.02 | 30 (12) | 27 (13) | 3 (7) |  |
| 0.03 | 6 (2) | 5 (2) | 1 (2) |  |
| 0.05 | 2 (1) | 2 (1) | 0 (0) |  |
| 0.06 | 1 (0) | 1 (0) | 0 (0) |  |
| Percentage of eosinophils, Median (IQR) | 0.1 (0, 0.4) | 0.2 (0.04, 0.52) | 0.05 (0, 0.18) | < 0.001 |
| Eosinophils count, Median (IQR) | 0.01 (0, 0.02) | 0.01 (0, 0.03) | 0 (0, 0.01) | 0.002 |
| INR, Median (IQR) | 1.02 (0.98, 1.07) | 1.02 (0.98, 1.06) | 1.04 (0.98, 1.1) | 0.189 |
| Urea nitrogen, Median (IQR) | 4.1 (3.23, 5.75) | 3.95 (3.1, 5) | 6.85 (5, 9.47) | < 0.001 |
| Uric acid, Median (IQR) | 252 (200.25, 310) | 248 (195.5, 306.25) | 302 (225, 428) | 0.001 |
| Average hemoglobin concentration, Median (IQR) | 320 (315, 326.75) | 320 (314.75, 326) | 322 (316, 327) | 0.613 |
| Average hemoglobin amount, Median (IQR) | 30 (29, 31) | 30 (29, 31) | 29 (28.25, 31) | 0.38 |
| Total carbon dioxide, Median (IQR) | 23.6 (21.8, 25.48) | 23.65 (22.17, 25.33) | 22.1 (20.3, 25.65) | 0.068 |
| Total bile acid., Median (IQR) | 5 (3.12, 7.7) | 4.65 (3.18, 7.35) | 6.15 (2.78, 8.23) | 0.282 |
| Total bilirubin., Median (IQR) | 8.3 (6.3, 10.3) | 7.85 (6.07, 9.93) | 10.3 (8.33, 12.63) | < 0.001 |
| Total protein., Median (IQR) | 66.3 (63.5, 70.6) | 66.2 (63.5, 70.62) | 66.7 (64.25, 69.72) | 0.943 |
| chlorine, Median (IQR) | 103 (101, 106) | 103 (101, 106) | 101.5 (98.25, 105.75) | 0.068 |
| APTT, Median (IQR) | 31.95 (28.7, 35.5) | 32 (28.78, 35.42) | 31.7 (28.63, 36.1) | 0.972 |
| Percentage of lymphocyte, Mean ± SD | 21.78 ± 10.82 | 23.13 ± 10.03 | 15.26 ± 12.2 | < 0.001 |
| Lymphocyte count, Median (IQR) | 0.94 (0.72, 1.21) | 0.98 (0.77, 1.26) | 0.74 (0.41, 0.96) | < 0.001 |
| globulin, Median (IQR) | 30.7 (27.52, 33.18) | 30.1 (27.17, 32.82) | 32.65 (30.2, 35.68) | < 0.001 |
| Ratio of leukocyte to globulin, Median (IQR) | 1.21 (1.04, 1.39) | 1.23 (1.07, 1.41) | 1.05 (0.86, 1.2) | < 0.001 |
| leukocyte, Median (IQR) | 4.6 (3.5, 6.27) | 4.5 (3.4, 5.8) | 5.6 (4.08, 9.07) | 0.004 |
| albumin, Mean ± SD | 36.35 ± 5.07 | 36.99 ± 4.99 | 33.29 ± 4.32 | < 0.001 |
| Direct bilirubin, Median (IQR) | 3.2 (2.5, 4.18) | 3 (2.5, 3.9) | 4.3 (3.32, 6.15) | < 0.001 |
| Alkaline phosphatase, Median (IQR) | 57 (46.25, 70) | 56 (46, 69) | 68 (50, 107.75) | 0.003 |
| phosphorus, Median (IQR) | 1 (0.87, 1.13) | 1 (0.9, 1.12) | 1.03 (0.84, 1.2) | 0.784 |
| erythrocytes, Median (IQR) | 4.33 (3.98, 4.71) | 4.33 (4.03, 4.71) | 4.32 (3.93, 4.62) | 0.551 |
| Width of RBC volume distribution, Median (IQR) | 12 (11.7, 12.5) | 12 (11.6, 12.3) | 12.4 (11.9, 13.07) | 0.005 |
| hematocrit, Median (IQR) | 39.9 (36.9, 42.77) | 39.9 (36.98, 43.1) | 39.85 (36.2, 41.1) | 0.625 |
| Average volume of RBC, Median (IQR) | 93 (90, 95) | 93 (90, 95) | 91.5 (90, 96) | 0.695 |
| fibrinogen, Median (IQR) | 4.19 (3.64, 4.93) | 4.21 (3.58, 4.92) | 4.09 (3.67, 5.06) | 0.632 |
| myoglobin, Median (IQR) | 53.39 (38.05, 93.75) | 50.6 (36.43, 79.53) | 125.28 (68.84, 249.06) | < 0.001 |
| creatinine, Median (IQR) | 68.5 (55.9, 84.67) | 67.1 (54.4, 82.62) | 71.4 (63.6, 105.7) | 0.01 |
| Creatine kinase, Median (IQR) | 94.5 (57.25, 178.25) | 87 (57, 154) | 158 (80, 359.25) | 0.005 |
| Creatine kinase isoenzyme, Median (IQR) | 12 (9, 16) | 11 (9, 15) | 17 (11.25, 22) | < 0.001 |
| Cystatin C, Median (IQR) | 0.9 (0.71, 1.07) | 0.88 (0.7, 1.02) | 1.12 (0.86, 1.52) | < 0.001 |
| Lipoprotein A, Median (IQR) | 93.55 (57, 154.45) | 92.45 (56.15, 154.15) | 95.05 (61.72, 152.45) | 0.803 |
| Platelet distribution width, Median (IQR) | 16.2 (15.9, 16.4) | 16.1 (15.9, 16.4) | 16.2 (16.1, 16.5) | 0.004 |
| Platelet hematocrit, Median (IQR) | 0.18 (0.14, 0.22) | 0.18 (0.14, 0.23) | 0.15 (0.12, 0.21) | 0.081 |
| Average platelet volume., Median (IQR) | 9.9 (9.2, 10.5) | 9.8 (9.2, 10.43) | 10.35 (9.57, 10.8) | 0.015 |
| Total platelet count., Median (IQR) | 179 (137.5, 236.75) | 184 (143.75, 240.5) | 146.5 (119.25, 199.5) | 0.011 |
| Serum magnesium., Median (IQR) | 0.82 (0.74, 0.91) | 0.8 (0.74, 0.89) | 0.84 (0.75, 0.99) | 0.188 |
| Blood glucose, Median (IQR) | 5.85 (4.99, 7.28) | 5.71 (4.98, 7) | 6.66 (5.31, 8) | 0.016 |
| hemoglobin., Median (IQR) | 127.5 (119, 137.75) | 127.5 (119, 138) | 127.5 (119, 135) | 0.98 |
| Glutamic pyruvic transaminase., Median (IQR) | 27 (19, 40.75) | 27 (18, 40) | 31.5 (22.25, 54.25) | 0.188 |
| Glutamic oxaloaminase, Median (IQR) | 33 (24.25, 46) | 31 (24, 44) | 44.5 (31.25, 76) | < 0.001 |
| hsC-reactive protein., Median (IQR) | 10 (10, 10) | 10 (10, 10) | 10 (10, 10) | 0.002 |
| Apolipoprotein A1, Median (IQR) | 0.98 (0.87, 1.11) | 1.02 (0.88, 1.12) | 0.92 (0.79, 1.06) | 0.002 |
| Apolipoprotein B, Median (IQR) | 0.77 (0.62, 0.9) | 0.77 (0.62, 0.9) | 0.8 (0.63, 0.92) | 0.857 |
| calcium, Median (IQR) | 2.1 (2.04, 2.18) | 2.11 (2.05, 2.18) | 2.06 (1.98, 2.14) | 0.005 |
| sodium, Median (IQR) | 142 (139, 144) | 142 (139.75, 144) | 140.5 (137, 145) | 0.351 |
| potassium., Median (IQR) | 3.9 (3.5, 4.3) | 3.85 (3.5, 4.3) | 3.95 (3.42, 4.54) | 0.621 |
| Indirect bilirubin., Median (IQR) | 4.7 (3.5, 6.27) | 4.6 (3.5, 6.1) | 5.35 (4.25, 6.83) | 0.105 |
| procalcitonin, Median (IQR) | 0.05 (0.05, 0.09) | 0.05 (0.05, 0.05) | 0.15 (0.06, 0.32) | < 0.001 |
| Hepatitis C antibody, Median (IQR) | 0.07 (0.05, 0.09) | 0.07 (0.05, 0.09) | 0.06 (0.05, 0.11) | 0.943 |
| HBeAb, Median (IQR) | 1.43 (0.86, 1.7) | 1.41 (0.83, 1.7) | 1.46 (0.94, 1.77) | 0.476 |
| HBeAg, Median (IQR) | 0.45 (0.42, 0.5) | 0.45 (0.42, 0.51) | 0.46 (0.43, 0.48) | 0.726 |
| HBcAb, Median (IQR) | 3.52 (0.15, 7.57) | 3.26 (0.15, 7.48) | 4.85 (0.22, 7.77) | 0.583 |
| HBsAb, Median (IQR) | 48.6 (1.81, 260.06) | 51.48 (2.67, 273.47) | 36.57 (0.5, 128.77) | 0.277 |
| HBsAg, Median (IQR) | 0.01 (0, 0.02) | 0.01 (0, 0.02) | 0 (0, 0.01) | 0.025 |
| HIVAb, Median (IQR) | 0.1 (0.08, 0.12) | 0.1 (0.08, 0.12) | 0.1 (0.09, 0.12) | 0.259 |
| Immunoglobulin IgA, Median (IQR) | 2.38 (1.81, 3.19) | 2.3 (1.76, 3.04) | 3.12 (2.29, 3.58) | < 0.001 |
| Immunoglobulin IgG, Median (IQR) | 13.67 (11.4, 15.31) | 13.15 (11.38, 14.79) | 14.99 (13.12, 16.69) | 0.003 |
| Immunoglobulin IgM, Median (IQR) | 1.3 (0.9, 2) | 1.3 (0.9, 2.1) | 1.25 (0.82, 1.8) | 0.312 |
| TPAb, Median (IQR) | 0.05 (0.04, 0.06) | 0.05 (0.04, 0.06) | 0.05 (0.04, 0.06) | 0.702 |
| C3, Mean ± SD | 1.14 ± 0.21 | 1.14 ± 0.2 | 1.14 ± 0.22 | 0.987 |
| C4, Mean ± SD | 0.4 ± 0.13 | 0.4 ± 0.13 | 0.4 ± 0.14 | 0.834 |
| BNP, Median (IQR) | 20.77 (7.17, 46.45) | 16.59 (5.53, 37.68) | 46.86 (28.83, 81.9) | < 0.001 |
| TOnsetToAdm, Median (IQR) | 7 (6, 10) | 7.5 (6, 10) | 7 (5, 10) | 0.61 |
| CmbdRespir, n (%) |  |  |  | 0.069 |
| FALSE | 232 (94) | 195 (96) | 37 (88) |  |
| TRUE | 14 (6) | 9 (4) | 5 (12) |  |
| CmbdCircu, n (%) |  |  |  | < 0.001 |
| FALSE | 183 (74) | 162 (79) | 21 (50) |  |
| TRUE | 63 (26) | 42 (21) | 21 (50) |  |
| CmbdDigestive, n (%) |  |  |  | 0.488 |
| FALSE | 230 (93) | 192 (94) | 38 (90) |  |
| TRUE | 16 (7) | 12 (6) | 4 (10) |  |
| CmbdRenal, n (%) |  |  |  | 0.139 |
| FALSE | 238 (97) | 199 (98) | 39 (93) |  |
| TRUE | 8 (3) | 5 (2) | 3 (7) |  |
| CmbdHematology, n (%) |  |  |  | 1 |
| FALSE | 245 (100) | 203 (100) | 42 (100) |  |
| TRUE | 1 (0) | 1 (0) | 0 (0) |  |
| CmbdEndoMeta, n (%) |  |  |  | 0.405 |
| FALSE | 212 (86) | 178 (87) | 34 (81) |  |
| TRUE | 34 (14) | 26 (13) | 8 (19) |  |
| CmbdPsychiatry, n (%) |  |  |  | 1 |
| FALSE | 239 (97) | 198 (97) | 41 (98) |  |
| TRUE | 7 (3) | 6 (3) | 1 (2) |  |
| CmbdReproductive, n (%) |  |  |  | 0.016 |
| FALSE | 242 (98) | 203 (100) | 39 (93) |  |
| TRUE | 4 (2) | 1 (0) | 3 (7) |  |
| CmbdMuscle, n (%) |  |  |  | 0.431 |
| FALSE | 243 (99) | 202 (99) | 41 (98) |  |
| TRUE | 3 (1) | 2 (1) | 1 (2) |  |
| CmbdInfect, n (%) |  |  |  | 0.009 |
| FALSE | 237 (96) | 200 (98) | 37 (88) |  |
| TRUE | 9 (4) | 4 (2) | 5 (12) |  |
| CmbdTrauma, n (%) |  |  |  | 0.031 |
| FALSE | 238 (97) | 200 (98) | 38 (90) |  |
| TRUE | 8 (3) | 4 (2) | 4 (10) |  |
| CmbdSurgery, n (%) |  |  |  | 0.207 |
| FALSE | 175 (71) | 149 (73) | 26 (62) |  |
| TRUE | 71 (29) | 55 (27) | 16 (38) |  |
| CmbdOthers, n (%) |  |  |  | 0.406 |
| FALSE | 235 (96) | 196 (96) | 39 (93) |  |
| TRUE | 11 (4) | 8 (4) | 3 (7) |  |
| Weight, Median (IQR) | 60 (55, 70) | 60 (55, 70) | 63.5 (55.75, 66.5) | 0.518 |
| Height, Median (IQR) | 165 (160, 170) | 163 (160, 170) | 167 (160, 170) | 0.466 |
| SBP, Mean ± SD | 130.17 ± 16.8 | 129.51 ± 15.89 | 133.36 ± 20.6 | 0.259 |
| DBP, Median (IQR) | 80 (74, 88) | 80 (74, 88) | 80 (70, 88.5) | 0.825 |
| Temperature, Median (IQR) | 36.9 (36.5, 37.6) | 36.9 (36.5, 37.6) | 36.8 (36.6, 37.62) | 0.636 |
| Pulse, Median (IQR) | 82.5 (78, 92) | 82.5 (79.5, 90) | 83 (78, 104) | 0.396 |
| RR, Median (IQR) | 20 (19.25, 22) | 20 (19, 21) | 21.5 (20, 25) | < 0.001 |

Abbreviations: IQR: interquartile range; SD: standard deviation; APTT: activated partial thrombin time; DBP: diastolic blood pressure; SBP: systolic blood pressure; LDL: low density lipoprotein; HDL: high density lipoprotein; INR: international normalized ratio; TG: triglyceride; TC: total Cholesterol; TPAb: Treponema pallidum antibody; BNP: brain natriuretic peptide; HIVAb: human immunodeficiency virus antibody; hrcp I: high sensitive troponin I; RR: respiratory rate; Cmbd: comorbidity.

# eFigure 1. Heatmap showing the difference between survival and non-survival groups.

eFigure 1. Heatmap showing the difference between alive and died groups.

All values were normalized by standard deviation and centered by mean. Light red color indicates higher values and dark black indicates lower values.

Abbreviations: APTT: activated partial thrombin time; DBP: diastolic blood pressure; SBP: systolic blood pressure; LDL: low density lipoprotein; HDL: high density lipoprotein; INR: international normalized ratio; TG: triglyceride; TC: total Cholesterol; TPAb: Treponema pallidum antibody; BNP: brain natriuretic peptide; HIVAb: human immunodeficiency virus antibody; hrcp I: high sensitive cardiac troponin I; RR: respiratory rate.

# eFigure 2. Forward selection using the most frequent genes (variables).


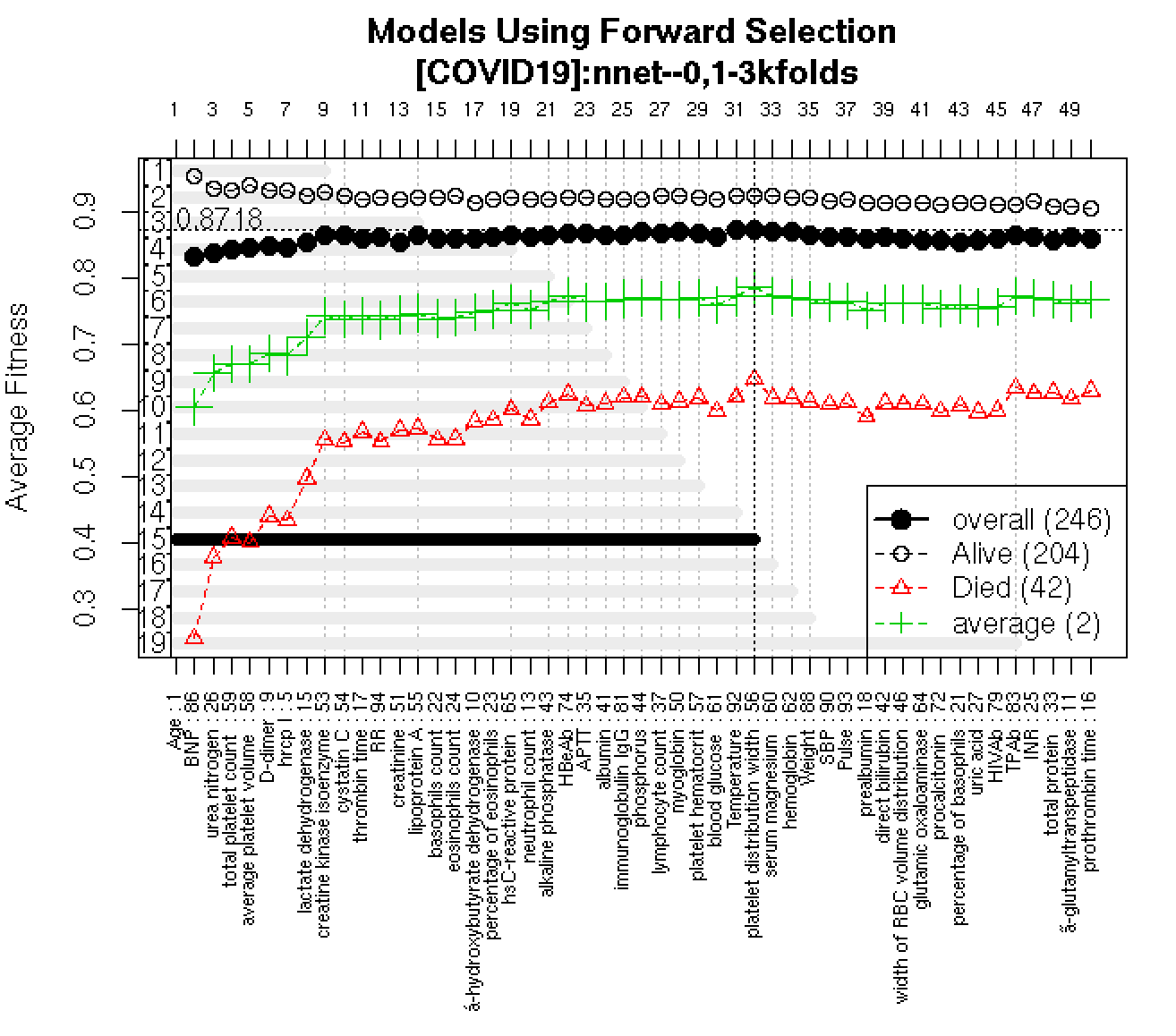


eFigure 2. Forward selection using the most frequent genes (variables). Horizontal axis represents the genes ordered by their rank. Vertical axis shows the classification accuracy. Solid line represents the overall accuracy (misclassified samples divided by the total number of samples). Coloured dashed lines represent the accuracy per class. There is one model resulted from the selection whose fitness value is maximum (black thick line), but 19 models were finally reported because their fitness is higher than 99% of the maximum.

# eFigure 3. Heatmap for NNet1 comprising 9 variables.

eFigure 3. Heatmap for NNet1 comprising 9 variables.

All values were normalized. The horizontal axis is the subject index, with the alive (black) versus died (red) status represented on the top color bar.

# eFigure 4. Heatmap for NNet1 comprising 32 variables.

eFigure 4. Heatmap for NNet1 comprising 32 variables.

All values were normalized. The horizontal axis is the subject index, with the alive (black) versus died (red) status represented on the top color bar.

# eFigure 5. Depiction of NNet1 in PCA space.

eFigure 5. Depiction of NNet1 in PCA space. Note that the first two components can best separate the alive and died groups.

# eFigure 6. Depiction of NNet2 in PCA space.

eFigure 6. Depiction of NNet2 in PCA space. Note that the first two components can best separate the alive and died groups.

# eFigure 7. Grid search hyperparameter tuning for NNet1 (A) and NNet2 (B).

eFigure 7. Grid search hyperparameter tuning for NNet1 (A) and NNet2 (B). The accuracy was evaluated with 5-fold cross validation. Hyperparameters included weight decay, hidden units and bag number.

# eFigure 8. Facetted heatmap-style visualization of all case-feature combinations for four selected patients.

eFigure 8. Facetted heatmap-style visualization of all case-feature combinations for four selected patients. The case numbers are shown in the horizontal axis and categorized features are shown in the vertical axis.
